# It's Time to Start Changing the Game: A 12-Week Workplace Team Sport Intervention Study

**DOI:** 10.1186/s40798-017-0099-7

**Published:** 2017-08-23

**Authors:** Andrew Brinkley, Hilary McDermott, Rachel Grenfell-Essam, Fehmidah Munir

**Affiliations:** School of Sport, Exercise and Health Sciences, National Centre for Sport and Exercise Medicine, Loughborough University, Loughborough, Leicestershire, LE11 3TU UK

**Keywords:** Communication, Fitness, Health, Quasi-experimental, Wellbeing, Workplace health, Workplace

## Abstract

**Background:**

A 12-week multi-team sport programme was provided to employees of a large services organisation and conducted in workplaces. This programme was used to investigate the short-term effect of regular sports team participation on individual employee and organisational health.

**Methods:**

A large services organisation participated in this study. Two regional worksites of office workers were assigned as the team sport (intervention) (*n =* 28 participants) or control (*n =* 20 participants) groups. The team sport sessions were underpinned by psychological behaviour change theory and consisted of weekly 1-h team sport sessions for 12 weeks. Measures of aerobic fitness, physical activity behaviour, group cohesion, interaction and communication, psychological wellbeing, health, anthropometrics and workplace experiences were recorded pre- and post-intervention. Data were analysed using a series of mixed ANOVAs.

**Results:**

After 12 weeks significant improvements were observed in VO_2_ max (+ 4.5 ± 5.8 ml/min kg, *P* < .002, *η* ^2^_p_ = .182), interpersonal communication within teams (+ 3%, *P* < .042, *η* ^2^_p_ = .087) and mean weekly physical activity duration (+ 154.74′, *P* < .002, *η* ^2^_p_ = .071) in the intervention group. A significant (*P* < .012, *η* ^2^_p_ = .130) effect on body composition was observed in the intervention group.

**Conclusions:**

Participation in team sport may be an effective method to improve the aerobic fitness and physical activity behaviour of employees, and promote interpersonal communication between colleagues. Individual health outcomes and social interactions have the capacity to influence the health of the organisation. The extent of which these findings are replicable across a scope of organisations should be examined objectively over the long term.

## Key Points

- This manuscript presents the first attempt to examine the efficacy of a workplace health promotion programme using a variety of team sports. This programme was the first of its kind implemented in a Financial Times and London Stock Exchange (FTSE 100) company and the first underpinned by self-determination theory.
- Past workplace team sport studies have examined markers of health without validated measures. This study tested the efficacy of workplace team sport across individual, social group and organisational health outcomes.
- This study provides support for the use of team sport to promote individual, social group and organisational health within a workplace setting.
- The findings of this study provide evidence for employers and occupational health teams considering implementing team sport into their workplaces.

## Background

Within Europe, almost half of working age adults are failing to meet physical activity (PA) guidelines [1]. Modifiable inactive behaviours are associated with non-communicable diseases (e.g. coronary heart disease and type-2 diabetes) and the prevalence of premature mortality [2]. An inactive workplace has been linked with diminished organisational health outcomes, increased sickness absence, reduced productivity and workplace engagement [3]. Workplace PA interventions can positively influence employee and organisational health [3]. Exercise/gym classes, walking, active-transport, educational training, active work stations and activity challenges have been utilised in the workplace with varying degrees of effectiveness [3]. However, research to date is yet to comprehensively examine the efficacy of sport and team sport on health outcomes [4]. Indeed, a further critique of workplace PA literature has been the failure to assess the efficacy of participation against social group and organisational health outcomes (e.g. communication, team performance, job satisfaction) [5].

Participation in workplace team sport has the capacity to improve individual, social group and organisational health outcomes [4]. Several randomised control trial (RCT) and quasi-experimental studies demonstrate that participation in competitive or non-competitive team sports can significantly improve cardiorespiratory fitness, musculoskeletal function [6–9] and psychological wellbeing [10]. These factors have the capacity to contribute to the reduced prevalence of non-communicable diseases associated with sickness absence, diminished productivity and all-cost mortality [2–5]. Qualitative studies suggest participation in team sports may also improve workplace relationships, communication and team cohesion [11–14].

Improvements in individual health and social group outcomes are known to contribute to the function of the organisation [3]. However, perhaps due to a limited level of funding and expertise, and the non-clinical community setting (i.e. the workplace), the research examining participation in team sport has been limited by the reliance on qualitative methods (e.g. focus groups and individual interviews) and interventions that lack strong theoretical underpinnings [4]. Researchers are yet to examine the impact of participation in workplace team sport on social group health outcomes (e.g. cohesion, communication, interpersonal relationships) with validated measures. Furthermore, the type of sport played is reported in most manuscripts but not the intensity it is played at, or the duration, volume and frequency of participation [11–14]. This challenges researchers in determining what ‘dose’ of team sport equals the benefits reported in the literature [4].

Participation in team sport is challenged by social comparisons with colleagues and superiors, the organisational culture and the facilities available within the workplace [15], and psychological barriers relating to autonomy, competence and relatedness [15, 16]. Self-determination theory (SDT) [17] suggests supporting people’s innate needs for autonomy (i.e. feeling free and fully volitional to engage in team sport), competence (i.e. feeling capable to complete a skill in team sport) and relatedness (i.e. feeling supported, understood and valued by a social group) through the provision of an activity (e.g. team sport) may promote wellbeing and autonomous motivation [14–16]. Good evidence indicates supporting basic needs is an effective behaviour change strategy within a workplace, sports and exercise setting [15–17]. For these reasons, workplace team sport interventions should be underpinned by behaviour change theories with a focus on the social environment [15–17].

This study evaluated the impact of a workplace team sport intervention (i.e. ‘Changing the Game’; CTG) underpinned by SDT. The primary intention of this study was to examine the impact of CTG on aerobic fitness (estimated VO_2_ max). Secondary intentions were to investigate participation in CTG’s impact on individual, social group and organisational health outcomes. These included subjective vitality, leisure-time PA, quality of life, occupational stress and fatigue, group cohesion, relationships with superiors and colleagues, communication, job satisfaction, individual and team job performance, and work engagement.

The intervention study was evaluated using a process evaluation underpinned by the RE-AIM approach [18]. This was conducted to evaluate the effectiveness of the intervention approach and its theoretical underpinning [18] and will be reported elsewhere (i.e. findings due for publication in late 2017^1^).

## Methods

### Design

**Fig. 1:**
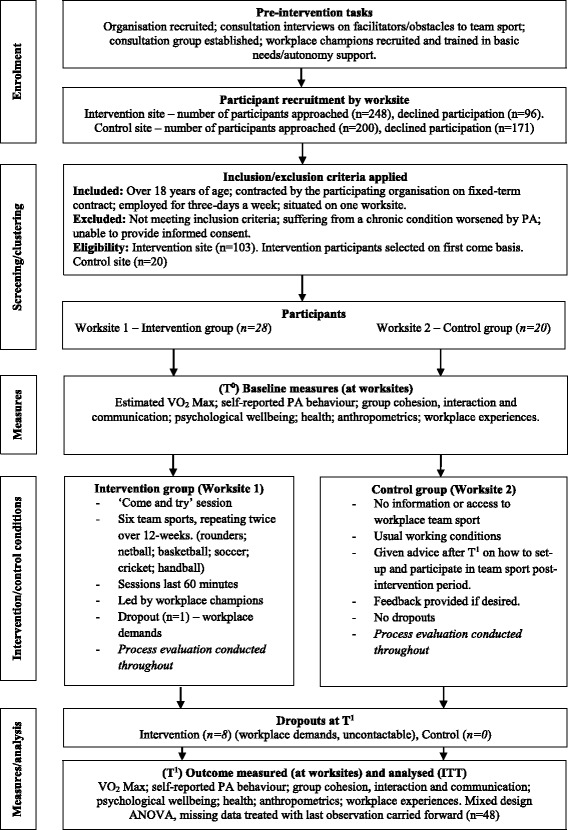
Schematic overview of ‘CTG’

The intervention was a 12-week non-randomised study (quasi-experimental design), which comprised two regional worksites from the same large service organisation (located an estimated 130 km apart). One worksite was assigned to the CTG (intervention group), while the other continued with normal working conditions (control group). Non-randomised intervention designs in health promotion are frequently adopted where feasibility and practicality issues challenge implementation [19]. In the current study, access to a local sport facility determined the intervention site. The participants were measured pre- (T^0^) and post- (T^1^) intervention at their respective workplaces. A schematic overview of the study’s design, recruitment and attrition rate is provided in Fig. 1. Ethical approval was obtained from Loughborough University’s Ethical Advisory Panel (Human Participants sub-committee). The study conforms to and was conducted in accordance with the Declaration of Helsinki.

### Intervention

CTG consisted of 12 weekly 1-h lunchtime moderate-intensity team sport sessions in an indoor sports hall (30 m × 18 m) located 400 m from the participating organisation. The sessions were funded by the researchers’ university and consisted of rounders (weeks 1 and 7), netball (weeks 2 and 8), basketball (weeks 3 and 9), soccer (weeks 4 and 10), cricket (weeks 5 and 11) and handball (weeks 6 and 12). The sessions included a 10-min warm-up and familiarisation period, and a 40-min game (breaks given when requested by participants). The sports were chosen by the research team due to their moderate-intensity, transferability of skills and adaptability. Evidence suggests sports with transferable skills may support the needs for competence [15–17]. The sessions were delivered by two female workplace champions trained by the first author in the basic rules of each sport and in supporting basic needs for autonomy, competence and relatedness in their sports participation (training information available on request from the corresponding author). Prior to the intervention participants were invited to take part in a taster session to help minimise perceptions of negative competence [15–17]. Throughout the CTG sessions, participants were encouraged to place an emphasis on having fun, to reflect on their individual and team skills development and social growth rather than competition and performance. Workplace champions discussed the potential benefits of participating in team sport with the participants who were able to opt out of any stage of the team sport sessions. Participants were given the opportunity to take ownership and alter any rule, skill or tradition associated with the sports as a team. This supportive leadership style can promote autonomous motivation and adherence to participation [15–17]. Adaptations made to the rules of each sport (e.g. removing double dribbles in basketball) has the capacity to support a participant’s need for competence, provide self-efficacy and promote the adoption of more autonomous forms of motivation (i.e. identified, integrated and intrinsic regulation) [17]. Needs for relatedness refer to the development and maintenance of interpersonal relationships with individuals (e.g. colleagues) and within workplace teams or groups [16, 17]. Relatedness may have been fostered through the social support, group identity and cohesion of colleagues and superiors playing team sports [15–17].

### Participants

A services organisation listed on the Financial Times and London Stock Exchange (FTSE) 100 located in the UK participated in this study. This organisation has a global workforce of 7048 employees (5080 operate in UK), across multiple worksites with employees in predominately office-based roles. This organisation was selected based on its size and structure and was recruited by email. A consultation group of employees, managers, workplace champions and employer representatives guided the intervention through a participatory approach [20]. The role of the consultation group was to advise on challenges specific to the participating organisation and discuss the implementation, delivery and evaluation of the programme within their organisation. Regular meetings were conducted with this working group throughout the intervention period.

Criterion-based sampling based on the inclusion and exclusion criteria was adopted.

Participants were recruited between May and June 2016, within the organisation through email, social media notifications and posters that outlined the purpose of the study. To ensure participants were motivated to participate, employees in both the intervention and control groups were recruited under the assumption they may be receiving team sport through their workplace. The intervention group was selected by access to a sports hall. More specifically, the intervention group was selected by its proximity to a sports hall, while the control group was selected from an isolated worksite. The control group did not have regular interactions with the intervention worksite. Employees interested in participating were sent an electronic or paper copy of the information sheet and informed consent form. Informed consent was obtained from all individual participants included in the study. Participants were screened and excluded from participation if they were under 18 years of age, were sub-contracted by another organisation, were contracted to work less than 3 days-a-week, had planned absences during the intervention period or were unable to provide informed consent. Following completion of the study, the control group was provided information on how to participate in workplace team sport.

Twenty eight participants (*n* = 8 females) in the intervention group were aged between 24 to 56 years (39.59 ± 9.11), and 20 participants (*n* = 12 females) in the control group age ranged between 24 to 64 years (40.75 ± 11.92). All participants worked within a team and represented a range of office-based job roles and levels of superiority (25% were in positions of superiority). The proportion of female participants (29%) reflects the proportion of females working within the organisation (i.e. 30% reported in 2016 annual report). Additional demographics are provided in Table 1.

**Table 1:** Participant demographic and baseline data (T^0^)

	Group				Sig
	Team sport		Control		
	M	SD	M	SD	
Age	39.59	9.11	40.75	11.92	.708
Gender (male = M, female = F)	M = 20, F = 8		M = 8, F = 12		.030*
Body mass index (BMI) (T^0^)	27.71	4.49	26.28	5.09	.931
Tenure (months)	119.77	123.01	139.35	162.11	.640
Average working hours	38.74	7.15	34.65	4.96	.034*
Average number of teams	2	1.46	1.3	.73	.057
Average team size	9.29	6.68	11.45	9.11	.355
Number of superiors	18		10		.064
VO_2_ max (ml/min/kg)	41.32	12.29	37.40	9.26	.236
Total MET per week	2474.08	2880.50	3444.40	2882.92	.256
Group cohesion	61.48^▼^	13.48	59.40	13.81	.604
Relationship superiors	47.92	16.33	57.07	11.12	.036*
Relationship colleagues	51.25	10.09	52.08	15.51	.850
Communication	74.42	10.74	79.40^▼^	11.71	.136
Subjective vitality	71.41	12.73	70.86	11.45	.878
Quality of life	68.57	16.51	74.42	16.69	.234
Stress	64.95	19.78	71.87	15.37	.198
Occupational fatigue	66.57	25.35	76.82^▼^	21.15	.147
Job satisfaction	75.51	9.41	75.71	10.46	.944
Job performance	82.75	9.38	83.57	10.44	.781
Team performance	63.27	14.70	68.29	15.11	.256
Work engagement	71.23	15.28	66.04	14.77	.246

Significant interactions indicated with *P < .05. Non-normally distributed data is indicated with ▼

### Measures

#### Primary Outcome Measure

Estimated maximal oxygen consumption (i.e. VO_2_ max) was recorded through a Chester step test (CST) [21]. The CST was conducted in accordance with a validated protocol [21]. Participants stepped on/off a 30-cm high exercise step until 80% MHR. Exercise heart rate was measured by a Polar™ T31 monitor and watch, and perceived exertion was indicated with the 15-point Borg scale [22]. The CST offers a feasible and ecologically valid means to examine maximal oxygen uptake with working age adults in community settings without discouraging participants prior to the intervention [23]. The CST was selected based on its re-test reliability [23]. A recent review [23] concluding the CST offers the most valid estimation of maximal oxygen uptake when compared to other step test protocols. The CST has correlated strongly (*r* = 0.92) with the output of VO_2_ max gas analysis conducted on a maximal oxygen usage treadmill test [21].

#### Secondary Outcome Measures

To determine body mass index (BMI) (kg/m^2^), height was measured to the nearest millimetre by a Leicester Height measure™, while weight in kilograms was measured by a Marsden™ M550 GP digital scale. The International PA Questionnaire ‘Short Form’ (IPAQ) estimated the total metabolic equivalent of task (MET) [24]. A self-report diary provided the day-to-day variation in frequency and duration of PA over the intervention period. PA diaries have strongly correlated with the output of objective measures of PA on indicators of duration (*r =* 0.82) [25]. Sub-scales from the Copenhagen Psychosocial Questionnaire-II were used to measure social cohesion and interpersonal relationships within the workplace [26]. Interpersonal communication within workplace teams was captured using five items reported in another study (Cronbach alpha = .95) [27]. Wellbeing was assessed using the Subjective Vitality Scale [28]; quality of life was measured with the Satisfaction with Life Scale [29]; the Perceived Stress Scale [30] measured self-reported stress; and occupational fatigue was assessed with the Need for Recovery Scale [31]. Job satisfaction was assessed with the Single-Item of Job Satisfaction [32]. Perceptions of individual job performance (four items) were measured on 5-point Likert scale [33]. Team performance was measured with the ‘team effectiveness’ sub-scale of the Aston Team Performance Inventory [34]. The Utrecht Work Engagement Scale (short form) captured work engagement [35]. All measures using scales were delivered in paper form and completed by hand.

### Data Analysis

A meta-analysis found medium Cohen’s effect sizes ranging from *d* = .47 to .57 for objectively estimated assessments of VO_2_ max [5]. A power calculation using G*Power [36] indicated 36 participants were required to observe a medium effect in the primary outcome, where *f* = .25, power is .95 and the error of probability is set at .05. A 35% attrition rate was applied. Analysis was conducted using IBM SPSS Statistics version 23, and *P* < .05 was considered statistical significant. Outliers were winsorized to the nearest non-outlying value. Self-reported items are represented with 0–100 scores (i.e. 0 is unfavourable). Normality was assessed through a Shapiro-Wilk test. Typically, data were normally distributed; however, where this is not the case, this is indicated in tables with a ^▼^. The magnitude of change is represented by a 95% confidence interval. Differences at T^0^ were assessed using a series of independent sample *t* tests. The primary and secondary outcomes were assessed using mixed design ANOVAs under the intention-to-treat principle [37]. Missing data were treated with the last observation carried forward method. For clarity, data were also examined with a series of mixed design ANCOVAs controlling for T^0^ data, gender, age, BMI and average working hours. These returned no contrasting findings. Significant findings were followed up with paired sample *t* tests. Data is represented by mean ± standard deviation.

## Results

### Observations at Baseline (T^0^)

Participant demographic data at baseline is presented in Table 1. The number of participants differed on a week-by-week basis (between 12 and 27 participants). One participant dropped out without attending any sessions. A series of independent sample *t* test found that at T^0^ the intervention and control groups did not significantly differ on the primary outcome of estimated VO_2_ max, or on any of the secondary outcomes apart from relationships with superiors (*P* < .036). The groups did not significantly differ in age, tenure, average team size, number of workplace teams or superiority. However, the groups did significantly differ on gender (*P* < .03) and average hours worked (*P* < .034). More specifically, 29% of the intervention group were female, while 60% of the control group were female. The control group worked 4.0907 h less per week than the intervention group. These were controlled for in the subsequent analysis.

### Main Analysis

#### Primary Outcome

**Fig. 2:**
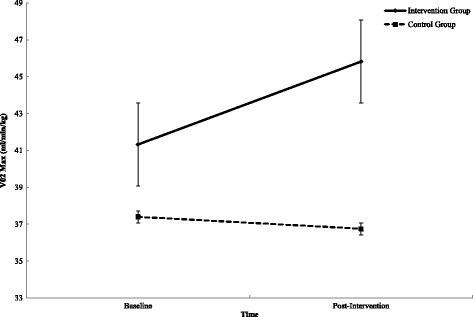
Interaction effect between T^0^ and T^1^ for intervention and control group on estimated VO_2_ max (ml/min/kg). Error bars represent standard error

Participation in workplace team sport significantly improved estimated VO_2_ max (*P* < .002, *η* ^2^_p_ = .182), when compared to the control group (see Table 2). A mixed design ANOVA captured a group × time interaction for mean estimated VO_2_ max (see Fig. 2). Follow-up paired samples *t* test observed a significant (*P* < .0001, *d* = .774) increase in estimated VO_2_ max of 4.5 ± 5.80 ml/min/kg (95% CI 2.248–6.752) in the intervention group. However, a non-significant reduction (*P* < .568, *d* = .129) of .65±5.00 ml/min/kg (95% CI − 2.299–1.694) was observed in the control group.

**Table 2:** Individual health outcomes for the team sport (intervention) and control groups assessed at baseline (T^0^) and at the end of the intervention (T^1^)

	T^0^		T^1^		*F* statistic		
	M	SD	M	SD	Group × time	Time	Group
VO_2_ max (ml/min/kg)	39.68	11.19	42.04	10.34	10.258**	5.733*	4.983*
Team sport	41.32	12.29	45.82	9.06			
Control	37.40	9.26	36.75	9.87			
Total MET per week	2878.38	2882.92	2830.58	2519.07	.472	.007	1.110
Team sport	2474.08	2880.50	2678.18	2282.26			
Control	3444.40	2882.92	3283.94	2840.80			
Subjective vitality	71.18	12.09	72.91	11.59	.437	.169	.919
Team sport	71.41	12.73	72.40	13.39			
Control	70.86	11.45	73.62	8.74			
Quality of life	71.01	16.67	70.41	17.05	.136	.493	.492
Team sport	68.57	16.51	69.69	16.91			
Control	74.42	16.69	71.24	17.63			
Stress	67.97	18.23	69.66	18.81	1.298	.358	.388
Team sport	64.95	19.78	68.97	19.72			
Control	71.87	15.37	70.62	17.92			
Occupational fatigue	70.84	24.00	74.06	25.36	.025	2.133	2.449
Team sport	66.57	25.35	69.49	27.90			
Control	76.82^▼^	21.15	80.45	20.27			
BMI (kg/m^2^)	27.12	4.75	27.58	4.42	6.788*	13.091***	.608
Team sport	27.71	4.49	27.86	4.41			
Control	26.28	5.09	27.20	4.52			

Significant interactions indicated with *P < .05, **P < .01, ***P < .001. Non-normally distributed data is indicated with ▼

#### Secondary Outcomes

**Fig. 3:**
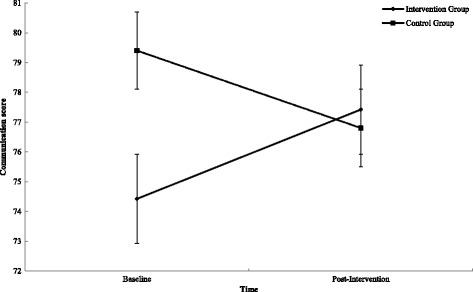
Interaction effect between T^0^ and T^1^ for intervention and control group on interpersonal team communication. Error bars represent standard error

A mixed design ANOVA revealed a significant group × time interaction on PA duration (minutes) (*P* < .002, *η* ^2^_p_ = .071) (see Table 2). The intervention group participated in significantly (*P* < .006) more PA per week than the control group (154.74 min) (95% CI 47.36–261.85). A mixed design ANOVA detected a significant group × time interaction on communication (*P* < .042, *η* ^2^_p_ = .087) (see Table 3). A follow-up paired samples *t* test observed a non-significant improvement (*P* < .85, *d* = .337) of 3.0 in interpersonal communication within workplace teams (95% CI − .446–6.446) in the intervention group and a non-significant (*P* < .235, *d* = .274) decrease of 2.6 (95% CI − 7.033–1.833) in the control group (see Fig. 3). A significant interaction was identified on relationships with colleagues over time only (*P* < .045, *η* ^2^_p_ = .085) (see Table 3). Further, a mixed design ANOVA captured a group × time interaction for mean BMI (*P* < .012, *η* ^2^_p_ = .130) (see Table 2). A follow-up paired samples *t* test observed a minor non-significant (*P* < .203, *d* = .246) increase in BMI of .146 ± .593 kg/m^2^ (95% CI .0837–.3765) in the intervention group. However, a significant (*P* < .008, *d* = .658) increase in BMI of .920 kg/m^2^ (95% CI .2659–1.574) was observed in the control group. All other secondary outcome variables were non-significant for group, time or group × time (see Tables 2, 3 and 4).

**Table 3:** Social group outcomes for the team sport (intervention) and control groups at baseline (T^0^) and at the end of the intervention (T^1^)

	T^0^		T^1^		*F* statistic		
	M	SD	M	SD	Group × time	Time	Group
Group cohesion	60.61	13.51	62.81	12.43	.014	2.018	.417
Team sport	61.48	13.48	63.83	11.81			
Control	59.40	13.81	61.39	13.42			
Relationship superiors	51.73	14.97	51.02	14.19	3.067	.490	2.514
Team sport	47.92	16.33	49.80	15.44			
Control	57.07	11.12	52.73	12.40			
Relationship colleagues	51.60	12.49	48.10	13.95	1.220	5.006*	.091
Team sport	51.25	10.09	49.32	10.92			
Control	52.08	15.51	46.39	17.50			
Communication	76.50	11.33	77.16	11.66	4.386*	.022	.495
Team sport	74.42^▼^	10.74	77.42^▼^	12.03			
Control	79.40	11.71	76.80^▼^	11.43			

Significant interactions indicated with *P < .05. Non-normally distributed data is indicated with ▼

**Table 4:** Organisational health outcomes for the team sport (intervention) and control groups at baseline (T^0^) and at the end of the intervention (T^1^)

	T^0^		T^1^		*F* statistic		
	M	SD	M	SD	Group × time	Time	Group
Job satisfaction	75.59	9.75	77.08	8.19	1.906	.737	.556
Team sport	75.51	9.41	78.57	9.11			
Control	75.71	10.46	75.00	6.34			
Job performance	83.22	9.91	83.33	9.96	.687	.004	.597
Team sport	83.57	10.44	84.64	9.51			
Control	82.75	9.38	81.50	10.52			
Team performance	65.36	14.92	64.83	13.60	.181	.166	.267
Team sport	63.27	14.70	63.30	14.80			
Control	68.29	15.11	66.97	11.74			
Work engagement	69.06	15.13	67.30	15.99	.631	1.780	2.062
Team sport	71.23	15.28	70.53	15.77			
Control	66.04	14.77	63.30	15.72			

Non-normally distributed data is indicated with ▼

## Discussion

### Summary of Findings

This non-randomised intervention study examined the impact of participating in workplace team sport upon a primary outcome of estimated VO_2_ max and secondary outcomes of individual, social group and organisational health. To our knowledge, this intervention study represents the first attempt to examine the impact of a multi-team sport programme within a workplace setting on multiple indicators of health.

A 10.32% increase in VO_2_ max was observed in the intervention group. Other intervention studies examining the impact of workplace soccer on aerobic fitness have observed improvements of 5% [8, 9]. Improvements in VO_2_ max are attributable to the prolonged exposure to high-intensity PA (HIPA) [9, 38]. HIPA improves cardiac output and skeletal muscle oxidation and capillarisation [8, 9, 38]. Decreased VO_2_ max (− 1.75%) in the control group may be explained by the sedentary job roles performed by these employees [2]. A previous review of research with intervention designs suggests sedentary behaviour has a detrimental impact on cardiac output [2].

Team sport may have a protective effect on BMI. Increases in the control group’s BMI (3.5%) may be attributable to the increased plasma glucose, triglycerides, lipoprotein and waist circumference associated with sedentary job roles [2]. Increases in energy expenditure in the intervention group may have caused participants to compensate by increasing nutritional intake and therefore BMI (.54%) [39]. This finding should be treated with caution given the absence of an RCT design, biomarkers, DEXA scans, skinfolds and nutritional intake. Adopting these measures may draw firmer conclusions.

Non-randomised (quasi-experimental) designs are however robust [18]. An RCT design may have been unethical, unfeasible or unrealistic to promote health within this workplace setting [18]. Likewise, quasi-experimental designs are effective at evaluating the efficacy of interventions and may be preferred due to feasibility, and the practical challenges of conducting a controlled design [18].

CTG did improve interpersonal communication within teams over time (3% in intervention group). This finding provides the first empirical support for several qualitative studies exploring the benefits of participation in workplace team sport [10–13]. Improvements in communication may be explained by the time employees spent together participating in an activity of common interest with shared goals [10]. Qualitative evidence indicates employees learn more about their colleagues’ communication style in a non-work environment with shared goals [10–13].

The employees in this study did not exclusively participate with their day-to-day colleagues, and therefore, the effect on working relationships, cohesion and team productivity may have been confounded. Future investigations examining workplace team sport may consider re-examining social group outcomes with a cluster RCT design whereby individual work teams are clustered into study arms. Although markers of individual health outcomes (e.g. psychological wellbeing) and organisational health outcomes (e.g. job satisfaction, productivity, work-engagement) did not significantly improve following participation, it would be unwise to suggest that team sport does not influence individual and organisational health. Rather, post hoc power analysis using G*Power [36] revealed most secondary outcome measures were unpowered (apart from quality of life) and unable to detect a significant interaction. Quality of life has shown to be a subjective state, and therefore, our findings may have been confounded by factors internal (e.g. salary, working practises) and external (e.g. personal relationships, lifestyle choices) [40] to the intervention. It may be of interest to researchers to re-examine markers of individual and organisational health with adequately powered outcome measures through day level (i.e. directly pre-post team sport) and over the long term (e.g. 6 months, 12 months).

### Limitations

The participants in this study represent a one large private services organisation. To prevent contamination, care was taken to select two isolated regional worksites. The groups did however differ in gender and average working hours at T^0^. Therefore, the study groups may not have been counterfactual. Given the absence of a RCT design and these differences between the groups, the results may have been confounded and caution should be applied when interpreting these findings. Although CTG was successful at recruiting female participants, only 29% of the intervention group were female. While this figure is proportionate to the number of females who work in the organisation (30% reported in 2016 annual report), researchers still may wish to understand why less female employees participate in team sport with their colleagues. Likewise, 89% of intervention group and 85% of control group participants reported meeting PA guidelines [1]. This figure is higher than the national average for working-age adults (67% of males, 55% of females). This may suggest workplace team sport attracts primarily active employees, rather inactive employees at risk of non-communicable illnesses and diseases. Future research may consider investigating the impact of workplace team sport with inactive employees.

A further limitation is the low number of employees initially recruited into a fully funded study from a large organisation. The current study’s process evaluation indicates recruitment over a 1-month period and ineffective communication strategy reduced the reach of the programme (process evaluation due for publication in 2017^1^). Intrapersonal, interpersonal, organisational and environmental factors may have facilitated participation or created obstacles for attendance [15]. Consistent with previous qualitative research, workplace demands, culture and practises challenged the consistent participation of employees [15]. Future research should continue to account for these factors using a participatory approach to implementation [20].

PA behaviour may be influenced by team sport participation over time. While the IPAQ and self-reported diary provide adequate psychometric properties, future research measuring PA would benefit from the addition of objective measures [41]. To maintain self-efficacy and competence, the current study avoided examination of sport intensity with heart rate monitors. While logical, this is a limitation of the study. Further, research may consider measuring intensity with heart rate monitors or perceived exertion (RPE) (e.g. the Borg Scale). Finally, data representing communication and occupational fatigue was not normally distributed and should be treated with caution. Future research may consider readdressing team sports impact on these outcomes.

## Conclusions

The current study examined the impact of a 12-week workplace team sport intervention on individual, social group and organisational health outcomes. Results indicate workplace team sport can improve aerobic fitness, PA behaviour and interpersonal communication within teams. These results suggest team sport may be an effective and viable form of health promotion within a workplace setting. Promoting forms of PA such as team sport within workplace settings is required to meet UK public health guidelines and reduce the financial and societal burden faced as attributable to an inactive population [42]. Therefore, it remains important to continue to understand why employees choose to participate in team sport and promote programmes which encourage participation. The current study suggests this may be achieved by promoting team sports which are supportive of autonomy, competence and relatedness. Researchers should consider testing the efficacy of a multi-team sport programme within a workplace setting over the long term with cluster RCT designs (i.e. randomise on workplace team level) and further objective measures of health (e.g. objective measures of physical activity, skinfold, DEXA scans, sickness absence).

## Footnotes

1 The process evaluation referenced in this article is due for publication in 2017. More specifically, this study is under-review at the time of acceptance of the current study. The process-evaluation provides further detail and information regarding the CTG’s reach, efficacy, adoption (e.g. participant adherence), implementation (e.g. theoretical underpinnings) and potential maintenance.

## References

[1] Townsend N, Wickramasighe K, Williams J, Bhatnager P, Rayner M (2015). Physical activity statistics 2015.

[2] Hamilton MT, Healy GN, Dunstan DW, Zderic TW, Owen N (2012). Too little exercise and too much sitting: inactivity physiology and the need for new recommendations on sedentary behaviour. Curr Cardiovasc Risk Reps.

[3] Batt ME (2009). Physical activity interventions in the workplace: the rationale and future direction for workplace wellness. Br J of Spor Medi..

[4] Brinkley A, McDermott H, Munir F (2017). What benefits does team sport hold for the workplace? A systematic review. J Spor Sci..

[5] Conn VS, Hafdahl AR, Cooper PS, Brown LM, Lusk SL (2009). Meta-analysis of workplace physical activity interventions. Am J Prev Med.

[6] Barene S, Holtermann A, Oseland H, Brekke OL, Krustrup P. Effects on muscle strength, maximal jump height, flexibility and postural sway after soccer and zumba exercise among female hospital employees: a 9-month randomised control trial. J Spor Sci. 2016; doi: 10.1080/026404414.2016.1140906 10.1080/02640414.2016.1140906 26849477

[7] Barene S, Krustrup P, Holtermann A. Effects of the workplace health promotion activities soccer and zumba on muscle pain, work ability and perceived physical exertion among female hospital employees. PLoS One. 2014;9(12) doi: 10.1371/journal.pone.0115059 10.1371/journal.pone.0115059 PMC4262471 25494175

[8] Barene S, Krustrup P, Brekke OL, Holtermann A (2014). Soccer and Zumba as health-promoting activities among female hospital employees: a 40-weeks cluster randomized intervention study. J Spor Sci..

[9] Barene S, Krustrup P, Jackman SR, Brekke OL, Holtermann A (2013). Do soccer and Zumba exercise improve fitness and indicators of health among female hospital employees? A 12-week RCT. Scand J Med Sci Spor.

[10] Scherrer P, Sheridan L, Sibson R, Ryan MM, Henley N (2010). Employee engagement with a corporate physical activity program: the global corporate challenge. Inter J Bus Stud.

[11] Joubert YT (2014). Sports contribution to open communication in a workplace: a qualitative study. J Psych Afr.

[12] Joubert YT, De Beer H (2010). Experiences of employees who participate in organisational team sport activities. J Emerg Trends Econo Manag Sci.

[13] Joubert YT, De Beer JJ (2011). Benefits of team sport for organisations. S Afr J Res Spor Phys Educ Recreat.

[14] Lee AL. Sports in the workplace do they pay? In: Diamant L, editor. Psychology of sports, exercise, and fitness: social and personal issues. New York: Hemisphere Publishing Corporation; 1991. p. 167–86.

[15] Brinkley A, Freeman J, McDermott H, Munir F (2017). What are the facilitators and obstacles to participation in workplace team sport? A qualitative study. AIMS Pub Heal.

[16] Gucciardi DF, Jackson B (2015). Understanding sport continuation: an integration of the theories of planned behaviour and basic needs theory. J Sci Med Spor.

[17] Deci EL, Ryan RM (2000). The “what” and “why” of goal pursuits: human needs and the self-determination of behavior. Psycho Inqu.

[18] Dzewaltowski DA, Glasgow RE, Klesges LM, Estabrooks PA, Brock E (2004). RE-AIM: evidence-based standards and a web resource to improve translation of research into practice. Ann Behav Med.

[19] Schelvis RMC, Oude Hengel KM, Burdorf A, Blatter A, Strijk JE, van der Beek AJ (2015). Evaluation of occupational health interventions using a randomized controlled trial: challenges and alternatives research designs. Scand J Work Environ Heal..

[20] Nielsen K, Randall R, Holten AL, González ER (2010). Conducting organisational-level occupational health interventions: what works?. Work Stress.

[21] Sykes K, Roberts A (2004). The Chester step test—a simple yet effective tool for the prediction of aerobic capacity. Physiother.

[22] Borg G (1990). Psychophysical scaling with applications in physical work and the perception of exertion. Scand J Work Environ Heal.

[23] Bennett H, Parfitt G, Davidson K, Eston R. Validity of submaximal step tests to estimate maximal oxygen uptake in healthy adults. Spor Med. 2015; doi: 10.1007/s40279-015-0445-1 10.1007/s40279-015-0445-1 26670455

[24] Craig CL, Marshall AL, Sjöström M, Bauman AM, Booth ML, Ainsworth BE (2003). International physical activity questionnaire: 12 country reliability and validity. Med Sci Spor Exerc.

[25] Connor SO, McCafferey N, Whyte E, Moran K (2016). The novel use of a SenseCam and accelerometer to validate training load and training information in self-recall training diary. J Spor Sci.

[26] Kristensen TS. A new tool for assessing psychosocial work environment factors: the Copenhagen psychosocial questionnaire. In M Hagberg, B Knave, L Lillienberg, H Westberg (Ed’s). x2001—expo and assess in Epidem and Pract. 2001; 210-213.

[27] González-Romá V, Hernández A (2014). Climate uniformity: its influence on team communication quality, task conflict, and team performance. J Appl Psych.

[28] Frederick CM, Ryan RM (1993). Differences in motivation for sport and exercise and their relations with participation and mental health. J Spor Behav.

[29] Diener E, Emmons RA, Larsen RJ, Griffin S (1985). The satisfaction with life scale. J Pers Assess.

[30] Cohen S, Kamarck T, Mermelstein R (1983). A global measure of perceived stress. J Heal Soci Behav.

[31] Veldhoven VM, Broersen S (2003). Measurement quality and validity of the “need for recovery scale”. Occup Environ Med.

[32] Dolbier CL, Webster JA, McCalister KT, Mallon MW, Steinhardt MA (2005). Reliability and validity of a single-item measure of job satisfaction. Am J Heal Promot.

[33] Munir F, Houdmont J, Clemes S, Wilson K, Kerr R, Addley K (2015). Work engagement and its association with occupational sitting time: results from the Stormont study. BMC Publ Heal.

[34] West MA, Markiewicz L, Dawson JF (2006). Aston team performance inventory: management set.

[35] Schaufeli WB, Bakker AB, Salanova M (2006). The measurement of work engagement with a short questionnaire a cross-national study. Educ Psych Meas.

[36] Faul F, Erdfelder E, Lang AG, Buchner A (2007). G*Power 3: a flexible statistical power analysis program for the social, behavioural, and biomedical sciences. Behav Res Meth.

[37] Elkins MR, Moseley AM. Intention-to-treat analysis. J Phys 2015; 61(3): 165-167. doi: 10.1016/j.jphys.2015.013. 10.1016/j.jphys.2015.05.013 26096012

[38] Krustrup P, Christensen JF, Randers MB, Pedersen H, Sundstrup E, Jakobsen MD (2010). Muscle adaptations and performance enhancements of soccer training for untrained men. Eur J Appl Physiol.

[39] King NA, Horner K, Hills AP, Byrne NM, Wood RE, Bryant E (2012). Exercise, appetite and weight management: understanding the compensatory responses in eating behaviour and how they contribute to variability in exercise-induced weight loss. Br J Spor Med.

[40] Gill DL, Hammond CC, Reifsteck EJ, Jehu CM, Williams RA, Adams MM (2013). Physical activity and quality of life. J Prev Medi Publ Heal.

[41] Kelly P, Fitzsimons C, Baker. Should we reframe how we think about physical activity and sedentary behaviour measurement? Validity and reliability reconsidered. Int J Behav Nutr Phys Act. 2016; doi: 10.1186/s12966-016-0351-4 10.1186/s12966-016-0351-4 PMC4772314 26931142

[42] Department of Health (2011). Start active, stay active: a report on physical activity for health from the four home countries’ chief medical officers.

